# Medication Safety in Intravenous Therapy: A Compatibility Study of Clonidine with Drugs Frequently Used in Intensive Care

**DOI:** 10.3390/pharmaceutics13010021

**Published:** 2020-12-24

**Authors:** Anna Katharina Koller, Sabine Krebs, Frank Dörje

**Affiliations:** Pharmacy Department, Erlangen University Hospital, 91054 Erlangen, Germany; sabine.krebs@uk-erlangen.de (S.K.); frank.doerje@uk-erlangen.de (F.D.)

**Keywords:** infusion therapy, medication safety, Y-site compatibility, clonidine hydrochloride, incompatibility, precipitation

## Abstract

The intravenous pharmacotherapy of critically ill patients is extremely challenging due to the high number of drugs administered. We therefore evaluated the physicochemical compatibility of combinations of clonidine with drugs frequently used in an intensive care unit setting. Amiodarone, dihydralazine, furosemide, levosimendan, metamizole, milrinone, urapidil, and verapamil were each prepared as binary combinations with clonidine at the standard low and high administration concentrations. Selected ternary combinations were also analyzed. Samples were examined for physical compatibility. To verify chemical compatibility in samples deemed either physically compatible or to exhibit uncertain results, the drug content was quantified using high-performance liquid chromatography. Admixtures of clonidine with amiodarone or furosemide proved to be physically incompatible, whereas mixtures with levosimendan and metamizole exhibited results, which were not clearly meeting the specification criteria for physical compatibility. Binary combinations of clonidine with dihydralazine, milrinone, urapidil, and verapamil were found to be physically compatible. Combinations with dihydralazine, levosimendan, metamizole, milrinon, urapidil, or verapamil were chemically compatible for the analyzed concentrations. Ternary admixtures of clonidine, metamizole, and urapidil; clonidine, metamizole, and verapamil; clonidine, urapidil, and verapamil were shown to be physicochemically compatible for the analyzed concentrations. These data suggest that clonidine can be coadministered with dihydralazine, levosimendan, metamizole, milrinone, urapidil, and verapamil. However, the concomitant administration of clonidine with amiodarone or furosemide is not recommended.

## 1. Introduction

Clonidine is an essential drug in the intensive care unit (ICU) setting. It is widely used due to its multiple beneficial effects as an antihypertensive and sedative agent. Consequently, this alpha-2 adrenoceptor agonist is of particular interest in critical care [1,2]. As an alpha-2-adrenoceptor selective imidazoline, it binds to the G-protein-coupled alpha-2a and alpha-2b receptors. Activation of alpha-2a-adrenceptors leads to anxiolytic and hypnotic-sedative effects and reduces central adrenergic activity, while activation of alpha-2b-adrenceptors leads to vasodilation, as well as increased hemodynamic stability and blood pressure reduction in patients [3,4,5].

Various studies and clinical reports have elucidated the clinical use of clonidine. It is utilized in the treatment of hypertension, hypertensive emergencies, atrial fibrillation, and the reduction of portal pressure. As a consequence, the intravenous application of clonidine has been approved by the FDA for the treatment of hypertension. 

Clonidine is also used to treat patients suffering from drug or alcohol withdrawal by decreasing muscle sympathetic activity and lowering catecholamine plasma concentrations [6]. In Germany, it is licensed for the i.v. treatment of tachycardia, tremor, perspiration, tachypnea, and agitation [7,8]. Moreover, both the American Society of Addiction Medicines and the Department of Veterans Affairs/Department of Defense recommend the use of clonidine for the treatment of opioid withdrawal management [9,10].

Clonidine is currently also used off-label as an effective coanalgetic and cosedative drug for pain management in critically ill or mechanically ventilated patients. This stems from studies showing that the application of clonidine may reduce the amount of anesthetics or benzodiazepines and opioids used if it is applied prior to surgical procedures [3,11]. Patients benefit from the administration of clonidine particularly during the perioperative period as its use does not lead to respiratory depression. A study by Ambrose et al. demonstrated that the coapplication of clonidine with midazolam in critically ill, ventilated pediatric patients led to a dose dependent sedation that was not accompanied by adverse effects on cardiovascular performance [12]. Furthermore, the SLEEPS-study showed that clonidine has major advantages as a safe and cost-effective sedative drug and leads to only minor withdrawal phenomena in comparison to midazolam [13]. A further study revealed that clonidine is applied at all stages of analgosedation as a cosedative in German hospitals. In total, 34% of hospitals used clonidine for sedation phases <24 h, 50% of hospitals for sedation phases between 24 and 72 h, and 53% of hospitals in the therapy of long-term sedation (>72 h). A total of 59% of hospitals applied clonidine as part of ventilator weaning medication [14]. Treatment with clonidine can be particularly beneficial when weaning patients who have been mechanically ventilated for a long period [15]. A national survey in British hospitals evaluated the sedation practice in ICUs. Clonidine was the second most commonly used drug for ventilator weaning and third most commonly used drug in patients with an expected ICU admission >24 h [16]. The application of clonidine can also result in less delirium in postoperative patients [17]. Consequently, clonidine has been included in the German S3-guideline for analgesia, sedation, and the management of delirium in critical care for more than 10 years [18].

The multiple positive clinical effects of clonidine render it an essential drug in an ICU setting, both for adults and for pediatric patients. However, in addition to the administration of clonidine, patients often simultaneously receive a number of other drugs via continuous infusion. The concomitant application of intravenous drugs via one catheter line is defined by the terms coapplication and coadministration. As the number of independent catheter lines of a central venous catheter is limited, the Y-site compatibility of the coadministered drugs is of utmost importance. An increasing number of applied drugs poses an inherent risk for safe pharmacotherapy [19]. Despite the widespread use of clonidine, very little information is available on the compatibility of clonidine with other drugs in international databases such as UpToDate^®^, Trissel’s Handbook on Injectable Drugs, Stabilis^®^, or international literature [20]. Veggeland identified this problem and published data on the visual compatibility of clonidine with 16 drugs, whereas Godwin et al. published compatibility data on the combination of clonidine and baclofen [21,22]. However, the information on the compatibility of clonidine remains incomplete and insufficient.

To shed light on the physicochemical compatibility of clonidine hydrochloride, this study analyzed its compatibility in concentrations of 4.5 and 15 µg/mL with eight drugs frequently used in German university hospitals. We identified the cardiovascular drugs amiodarone hydrochloride, dihydralazine mesilate, levosimendan, milrinone, urapidil, and verapamil; the diuretic furosemide; and the analgetic metamizole as highly important drugs in the intravenous therapy of critically ill patients. These drugs are regularly coadministered with clonidine in daily practice. In this study, the standard high and low concentrations of each of these drugs used in ICUs were determined before they were combined with clonidine hydrochloride. Furthermore, selected ternary combinations (clonidine hydrochloride, urapidil, and verapamil; clonidine hydrochloride, metamizole sodium, and urapidil; clonidine hydrochloride, metamizole sodium, and verapamil hydrochloride) were analyzed, since they are of high clinical relevance in the ICU setting. All samples were analyzed according to physical parameters of the admixtures over a time period of 24 h. Subsequently the drug content of physically compatible combinations or admixtures displaying results that did not clearly meet the predefined specifications was quantified using high-performance liquid chromatography (HPLC).

## 2. Materials and Methods

### 2.1. Sample Preparation

Drug combinations were generated by admixing the combination partners. Admixture preparation was performed by putting the carrier solutions into a colorless ethylene-vinylacetate infusion bag and adding the drug concentrates (Table 1). However, admixtures with dihydralazine mesilate were prepared in polyvinylchloride-free infusion bags due to the incompatibility of dihydralazine mesilate with materials containing polyvinylchloride. For the preparation of the drugs and the carrier solutions, Luer-Lock syringes identical to those used in the ICUs were employed. Samples were stored in the upright position. All admixtures were prepared at a total volume of 250 mL as a volume of 30 mL was needed to conduct the measurements for each time point.

The drug solutions of clonidine hydrochloride and the combination partners were added as detailed in Table 1. Combinations of clonidine hydrochloride with amiodarone hydrochloride, dihydralazine mesilate, furosemide sodium, levosimendan, metamizole sodium, milrinone, urapidil, and verapamil hydrochloride were generated. To obtain compatibility data for the ready to use drug clonidine hydrochloride 30 µg/mL produced by the Erlangen University Hospital, no other commercially available clonidine hydrochloride preparations were investigated. Table A1 in Appendix A shows the detailed composition of clonidine hydrochloride 30 µg/mL.

The experiments were conducted in duplicate, with the drugs being added to the bag in both possible orders. By combining the lowest and highest possible concentrations (low-low, low-high, high-low, high-high) in the investigation scheme, all relevant possible concentration ranges for stability testing were covered. Furthermore, the revision experiments were performed with drugs and carrier solutions of different batches. Admixtures were prepared at a 1:1 ratio, specifically the ratio employed in practice via a Y-injection site, and stored at room temperature in a temperature-monitored laboratory (22–24 °C) [23]. The infusion bags were not protected from light, with the exception of the preparations with amiodarone hydrochloride and metamizole sodium. As amiodarone hydrochloride and metamizole sodium are known to be highly light-sensitive, these infusion bags were stored in light protecting bags. Ternary combinations of clonidine with metamizole and urapidil; clonidine, metamizole, and verapamil; clonidine, urapidil, and verapamil were prepared in their standard high concentrations. All experiments were conducted according to existing guidelines for assessing chemical stability and compatibility [24,25,26].

Samples were drawn at specified time intervals after admixture (0, 0.5, 1, 2, 4, 6, and 24 h). A time period of 6 h was used to assess compatibility, while a time period of 24 h was used to evaluate stability of the drug admixtures. The time period of 24 h was chosen due to the fact that the majority of continuous infusions are discarded after 24 h owing to microbial stability as stated in product information. Part of each sample was immediately analyzed on the basis of visual and physical parameters. Two additional samples were drawn at the time intervals mentioned previously and were stored at −80 °C to prevent any possible drug degradation occurring before the later drug quantification. The technical devices used for this study were qualified and calibrated according to guidelines for good manufacturing practice.

### 2.2. Physical Analysis 

The samples were analyzed on the basis of visual characteristics, pH changes, photometrical changes at the absorption of 420 and 550 nm and the formation of subvisual particles (Table 2).

All admixtures were examined in the infusion bags by the unaided eye under fluorescent laboratory light in front of a black and white background. Any signs of visual incompatibilities, such as a change in color, precipitation, or gas formation, were recorded. Gas formation was defined as the appearance of visible gas bubbles over the reaction time.

In line with Monography 2.10.19 of the European Pharmacopoeia, the determination of subvisual particles for infusions with volumes greater than 100 mL was conducted using a particle counter [27]. 

A pH variation of more than 0.4 pH units was prespecified as the cut-off, representing a variation in the concentration of H_3_O^+^ ions by a factor of 2.5. The pH value constitutes a highly critical parameter for the stability of injectable drugs. During the manufacturing process, the pH value is adjusted to obtain the most stable drug preparation. A change in pH value caused by the addition of another drug may therefore exert a significant influence on the stability of a drug solution. International compatibility studies have used pH value variations ranging from 0.2 to 1.0 pH units [28,29,30].

The spectroscopic measurement was performed using an ultraviolet visible spectrophotometer to detect any indications of color change (420 nm) or haze (550 nm) over the relevant time period [29]. The admixtures of the carrier solutions were used as a reference. Admixtures were considered compatible if the absorption did not vary by more than 0.04 units at 420 nm or 0.01 units at 550 nm [29]. For admixtures containing levosimendan or metamizole, it was not appropriate to measure the variation of absorption. The product information of the yellow-colored levosimendan specifically states that any change in color over the time period does not result in any reduction in quality, while metamizole hydrolyzes to a colored degradation product [31].

### 2.3. Chemical Analysis 

Drug admixtures which either met the criteria for physical compatibility or which displayed results at the limits of specification were analyzed for chemical stability via HPLC to quantify the drug content. Samples which displayed subvisual particles at any time during the study were excluded from HPLC analysis, as were admixtures with obvious incompatibilities, such as a marked color change or a substantial change in pH value.

The HPLC methods applied for drug quantification are shown in Table A2. A Kinetex^®^ 2.6 µm Phenyl-Hexyl 100Å, 150 × 2.1 mm column was used for quantification. The eluent consisted of methanol and K_2_HPO_4_ buffer, pH 6.8. It was not possible to quantify clonidine, dihydralazine, and urapidil with this method. Therefore, methods adapted from the European Pharmacopoeia were optimized for the separation of the drugs from combination partners and degradation products. Each application was validated according to the ICH quality guideline Q2 “Validation of Analytical Procedures: Methodology” in terms of specificity, linearity, reproducibility, and precision [32]. The specificity was analyzed by treating four samples of clonidine with NaOH 0.1 M, HCl 0.1 M, H_2_O_2_ 30% or by thermal exposure. The chromatograms were evaluated for baseline resolution and the main peaks were clearly delimited to all degradation products. Efficient baseline resolution was achieved. A 70–130% range of the expected drug concentrations was measured to determine the linearity of the applied method. The linear regression was calculated from the mean values of the peak areas of three replicates and the correlation coefficient was calculated to evaluate the variation. The specification was set to a correlation coefficient of >0.99 and each method met the criteria. The precision and reproducibility were measured on three days. Three samples were analyzed on each day and the specification was set to a relative standard deviation of the mean peak area of ≤2.0%. The relative standard deviation of the peak areas complied with the specification for each method.

Prior to each analysis, a system suitability test was conducted: six replicates of drug solution to be investigated were injected and showed a relative standard derivation of less than 1%.

A calibration curve was measured in every run. Drug admixtures were considered chemically compatible if the concentrations of each drug remained between 90 and 110% of the original concentration [29,33,34,35,36,37,38]. As the samples were stored at −80 °C to prevent any drug reaction or degradation, prior to drug quantification via HPLC they were thawed under light protection at room temperature (22–24 °C). As part of the validation process it could be shown that no decrease caused by thawing in the drug concentration was detected.

## 3. Results

### 3.1. Physical Compatibility

Clonidine hydrochloride appeared to be physically compatible with the majority of the drugs tested (Table A3). The binary admixtures with dihydralazine mesilate, furosemide sodium, milrinone, urapidil, and verapamil hydrochloride remained clear, colorless, and free of gas formation over the time period of 24 h. Combinations of clonidine hydrochloride with levosimendan were bright yellow, and combinations with metamizole sodium also developed a slight yellow color. By contrast, a visible white precipitate formed in all combinations with amiodarone hydrochloride by 24 h.

Analysis for subvisual particles revealed subvisual particles in combinations of clonidine hydrochloride 4.5 µg/mL and amiodarone hydrochloride 3 mg/mL at 24 h. All other combinations of clonidine hydrochloride and amiodarone hydrochloride, including clonidine hydrochloride 15 µg/mL and amiodarone hydrochloride 3 mg/mL; clonidine hydrochloride 4.5 µg/mL and amiodarone hydrochloride 12 mg/mL; clonidine hydrochloride 15 µg/mL and amiodarone hydrochloride 12 mg/mL, displayed an increased quantity of subvisual particles not within the specifications immediately after admixture, as well as a visible, white precipitate after 24 h incubation time. Additionally, combinations of clonidine hydrochloride 4.5 µg/mL and amiodarone hydrochloride 12 mg/mL, and clonidine hydrochloride 15 µg/mL and amiodarone hydrochloride 12 mg/mL, showed a variation in absorption at 420 and 550 nm at 24 h incubation time. Consequently, the combinations of clonidine hydrochloride with amiodarone hydrochloride were classified as physically incompatible.

All combinations of clonidine hydrochloride 4.5 and 15 µg/mL with dihydralazine mesilate 0.25 and 0.75 mg/mL complied with specifications for physical compatibility at every time point and were therefore classed as physically compatible.

Admixtures of clonidine hydrochloride 4.5 µg/mL with furosemide sodium 0.64 mg/mL remained within specifications for physical compatibility, whereas combinations of clonidine hydrochloride 15 µg/mL and furosemide sodium 0.64 mg/mL displayed a major change in the pH value from 2 h incubation time onwards. Clonidine hydrochloride 4.5 µg/mL and furosemide sodium 5.34 mg/mL showed a major variation of the pH value at 6 h incubation time and admixtures of clonidine hydrochloride 15 µg/mL and furosemide sodium 5.34 mg/mL were not within specifications for the pH value at 4 h reaction time. As a result, the combinations of clonidine hydrochloride with furosemide sodium were classified as physically incompatible.

Both admixtures of clonidine hydrochloride 4.5 µg/mL and clonidine hydrochloride 15 µg/mL with levosimendan 12.5 µg/mL displayed a change in absorption at 420 nm at 0.5 h after admixture. As levosimendan is a bright yellow drug, this change of absorption at 420 nm could not be used to evaluate of physical compatibility.

Binary admixtures of clonidine hydrochloride 4.5 µg/mL and metamizole sodium 25 mg/mL showed a slight change in the pH value at 1 h reaction time. Furthermore, a change in absorption at 420 nm could be detected. Combinations of clonidine hydrochloride 15 µg/mL and metamizole sodium 25 mg/mL displayed a minor change in pH value at 1 h incubation time, as well as a change in absorption at 420 nm at 6 h. Both combinations of clonidine hydrochloride 4.5 µg/mL and clonidine hydrochloride 15 µg/mL with metamizole sodium 50 mg/mL displayed a minor pH value change and a change in absorption at 420 nm at 6 h reaction time. These results prevented any conclusions about the physical compatibility of clonidine hydrochloride and metamizole sodium from being drawn.

Admixtures of clonidine hydrochloride with dihydralazine mesilate, milrinone, urapidil, and verapamil hydrochloride remained within specifications and were classified as physically compatible.

Ternary combinations of clonidine hydrochloride 10 µg/mL, metamizole sodium 33 mg/mL, and urapidil 1.67 mg/mL as well as clonidine hydrochloride 10 µg/mL, metamizole sodium 33 mg/mL, and verapamil hydrochloride 0.33 mg/mL complied with the specifications concerning visual inspection, pH value, and subvisual particles. However, at a reaction time of 6 h the absorption at 420 nm was out of specification. Ternary combinations of clonidine hydrochloride 10 µg/mL, urapidil 1.67 mg/mL, and verapamil hydrochloride 0.33 mg/mL complied with the specifications throughout the 24 h time period (Table A4).

### 3.2. Chemical Compatibility

Drug combinations that were deemed to be physically compatible or that displayed results not clearly meeting the specifications for physical compatibility were quantified via HPLC to determine drug content. As all admixtures of clonidine hydrochloride with amiodarone hydrochloride and three out of four combinations of clonidine hydrochloride with furosemide sodium were classified as physically incompatible, they were not analyzed further.

Binary combinations of clonidine hydrochloride 4.5 µg/mL and clonidine hydrochloride 15 µg/mL with dihydralazine mesilate 0.25 mg/mL and dihydralazine mesilate 0.75 mg/mL complied with the specification throughout the allotted time range. Binary admixtures of clonidine hydrochloride 4.5 µg/mL and clonidine hydrochloride 15 µg/mL in combination with levosimendan 12.5 µg/mL exhibited a decline in levosimendan to 85% of the original quantity by the 24 h time point, whereas the clonidine concentration remained constant over time (Table A5). Combinations of clonidine hydrochloride 4.5 µg/mL and clonidine hydrochloride 15 µg/mL with metamizole sodium 25 mg/mL and metamizole sodium 50 mg/mL displayed a minor decline in metamizole quantity within the 24 h period. The clonidine concentration also decreased, though the concentration of both combination partners remained within specifications throughout the 24 h time range.

All admixtures of clonidine hydrochloride 4.5 µg/mL and clonidine hydrochloride 15 µg/mL with milrinone 0.1 mg/mL, urapidil 1 mg/mL, urapidil 2.5 mg/mL, and verapamil hydrochloride 0.5 mg/mL retained constant quantities of drug throughout the full 24 h period. Ternary combinations of clonidine hydrochloride 10 µg/mL, metamizole sodium 33 mg/mL, and urapidil 1.67 mg/mL showed a decline below the specification for urapidil at 24 h, while the amount of clonidine and metamizole decreased only slightly (Table A6). A slight decrease in metamizole was also evident in admixtures of clonidine hydrochloride 10 µg/mL, metamizole sodium 33 mg/mL, and verapamil hydrochloride 0.33 mg/mL. The drug concentrations in ternary combinations of clonidine hydrochloride 10 µg/mL, urapidil 1.67 mg/mL, and verapamil hydrochloride 0.33 mg/mL remained constant throughout the 24 h time period.

## 4. Discussion

In this study the physicochemical compatibility for clonidine hydrochloride in binary combinations with amiodarone hydrochloride, dihydralazine mesilate, furosemide sodium, levosimendan, metamizole sodium, milrinone, urapidil, and verapamil hydrochloride was evaluated. Furthermore, three ternary combinations of clonidine hydrochloride with metamizole sodium, urapidil, and verapamil hydrochloride were analyzed as these combinations were considered highly relevant for infusion therapy in ICUs. The applied methods for the compatibility testing proved to be valid and robust. Moreover, the technical devices used to conduct the analysis were qualified and calibrated according to guidelines for good manufacturing practice. This was the basis for the extensive analytical investigations.

Clonidine hydrochloride did not exhibit any signs of physical incompatibility in binary combinations with dihydralazine mesilate, milrinone, urapidil, and verapamil hydrochloride. Furthermore, the chemical analysis on the drug content of the combination partners demonstrated that the drug concentrations clearly met all specification criteria throughout the time period of 24 h. Consequently, these combinations were classed as physicochemically compatible (Table 3).

As admixtures with levosimendan showed a change of absorption at 420 nm, the physical compatibility could not be determined. The HPLC analysis revealed that the levosimendan content complied with the specification until 6 h. The admixture was classified as physicochemically compatible for 6 h only as it was not stable for 24 h [39]. 

All combinations of clonidine hydrochloride with metamizole sodium displayed a change in absorption at 420 nm of more than 0.04 units. As one hydrolysis product of metamizole sodium has a yellow color, the prespecified criteria are not applicable for the determination of the physical compatibility. However, a slight change in the pH value over time indicated a reaction in the drug admixture. This was caused by the hydrolysis of metamizole sodium. Nevertheless, the HPLC quantification showed that the drug content was within the present specification criteria in every admixture throughout the time range, despite a minor decline in metamizole sodium concentration caused by hydrolysis.

Combinations of clonidine hydrochloride with amiodarone hydrochloride exhibited formation of visual and subvisual particles and were therefore classified as physically incompatible admixtures. The appearance of such a precipitate may be due to the dilution of polysorbate, an ingredient of amiodarone hydrochloride infusion and required as a solubilizer for amiodarone hydrochloride. Particles pose a major risk for critically ill patients. In addition to the inadequate drug concentration reaching the patient, infused particles can be critical [40,41]. The resulting complications range from mild symptoms such as local redness, phlebitis, and thrombophlebitis to thrombosis and allergic reactions. More severe complications such as pulmonary embolism, severe allergic reactions, and the formation of an embolus can result in severe hepatic dysfunction, toxic shock, organ failure, or multiple organ failure and thus pose a lethal risk for the patient [42,43]. Admixtures of clonidine hydrochloride and furosemide sodium partially displayed variations in the pH values of the admixtures. This may be due to the acidic pH value of clonidine hydrochloride solution and the alkaline pH value of furosemide sodium solution. Existing publications describe an optimum pH value for clonidine hydrochloride as 4.0–7.0 whereas the pH value for furosemide sodium for injection should not fall below 7.0 [44,45]. The disparity in pH-optima for the two drugs may explain their incompatibility.

The ternary combinations of clonidine hydrochloride tested exhibited no signs of incompatibility. While the combination with metamizole sodium and urapidil showed a decrease of urapidil at 24 h, it complied with the specification criteria at the 6 h point. As one of the hydrolysis products of metamizole sodium is formaldehyde, the observed decline of urapidil could be caused by a reaction with formaldehyde. The amount of metamizole also decreased due to hydrolysis of metamizole. However, it remained within the specification criteria throughout the time range. Combinations of clonidine hydrochloride with metamizole sodium and verapamil hydrochloride appeared to be compatible, with all drug amounts complying with the specification criteria. The ternary combination of clonidine hydrochloride with urapidil and verapamil hydrochloride was also shown to be physicochemically compatible.

Clonidine did not display any sign of incompatibilities with the carrier fluids sodium chloride 0.9% and dextrose 5%, or the material of the infusion bags. This should be noted, as incompatibilities with carrier solutions or tubing and infusion bags may represent a risk for incompatibilities in daily practice. 

As international data on the compatibility of clonidine is currently incomplete, to date only one publication describes its compatibility with drug combinations investigated here. Veggeland previously described the visual compatibility of clonidine and verapamil in a study combining clonidine 18 µg/mL with 2.5 mg/mL. This combination was classified as visually compatible [21], correlating with the results of the present study.

It should be noted that the results of the compatibility study of clonidine hydrochloride in binary and ternary combinations are limited to the preparations and the concentrations used in this study and may not apply to the preparations of other manufacturers or different concentrations. However, to the best of our knowledge the drug concentration ranges tested in this study reflect current clinical application practice.

## 5. Conclusions

This study clearly illustrates the physicochemical compatibility of clinically relevant combinations of clonidine hydrochloride with commonly used drugs in the ICU setting. Our findings demonstrate that clonidine hydrochloride can be safely coadministered with dihydralazine mesilate, levosimendan, metamizole sodium, milrinone, urapidil, and verapamil hydrochloride for the analyzed concentrations, though it should not be administered together with amiodarone hydrochloride or furosemide sodium via one catheter line. Moreover, amiodarone hydrochloride carries a potentially major risk for precipitation if its solubilizer is significantly diluted and furosemide sodium leads to multiple incompatibilities due to its alkaline pH value. Future structural analysis, for example HPCL-MS, could be beneficial to identify possible degradation products of interest in more depth.

Clonidine’s beneficial effects as a potent coanalgetic and cosedative render it an indispensable drug in the treatment of critically ill patients in intensive care units. Through its multiple positive effects, clonidine helps to ensure a safe and effective i.v. pharmacotherapy at intensive care units for both adult and pediatric patients.

This novel data on the compatibility of clonidine broadens current knowledge of the safe coadministration of essential drugs in the ICU setting. It provides a basis for safer infusion regimens in daily clinical practice. The reported work is substantially contributing to a better medication safety in ICU medicine.

## Figures and Tables

**Table 1:** Drugs and standard concentrations tested for compatibility.

INN, Trade Name	Manufacturer	Lot	Concentration	Carrier Solution
Amiodarone hydrochloride, Cordarex^®^	Sanofi-Aventis GmbH, Frankfurt am Main, Germany	CY028, DY009	3.0–12.0 mg/mL	5% Dextrose injection
Clonidine hydrochloride 30 µg/mL	Pharmacy Department Erlangen University Hospital, Erlangen, Germany	021.17, 031.17, 032.17, 033.17, 052.17, 069.17, 004.18, 031.18, 058.18	4.5–15.0 µg/mL	0.9% Sodium chloride injection
Dihydralazine mesilate, Nepresol Inject^®^	Teofarma srl, Pavia, Italy	1603G167461, 1605G167462, 1703G178286	0.25–0.75 mg/mL	0.9% Sodium chloride injection
Furosemide sodium, Furosemid-ratiopharm^®^	Ratiopharm GmbH, Ulm, Germany	T00561, T10580A, T17400, V01925A	0.64–5.34 mg/mL	0.9% Sodium chloride injection
Levosimendan, Simdax^®^	Orion Pharma GmbH, Espoo, Finland	1671501, 1779857, 1733853	12.5 µg/mL	5% Dextrose injection
Metamizole sodium, Novaminsulfon-ratiopharm^®^	Ratiopharm GmbH, Ulm, Germany	S40432, T01008, T06694A, T30783A	25.0–50.0 mg/mL	0.9% Sodium chloride injection
Milrinone, Corotrop^®^	Sanofi-Aventis GmbH, Frankfurt am Main, Germany	CY013, 001DY, D2330	0.1 mg/mL	0.9% Sodium chloride injection
Urapidil, Urapidil 100 mg i.v. Carino^®^	CARINOPHARM GmbH, Elze, Germany	015491, 016118, 018428	1.0–2.5 mg/mL	0.9% Sodium chloride injection
Verapamil hydrochloride, Verapamil-ratiopharm^®^	Ratiopharm GmbH, Ulm, Germany	S41186, S20379, U02636A, U026364	0.5 mg/mL	0.9% Sodium chloride injection
Sodiumchloride 0.9%	Berlin-Chemie, Berlin, Germany	64070508, 71072508, 72074508, 72075508, 72078508, 72082508, 72085508, 81096508		
Dextrose 5%	B. Braun, Melsungen, Germany	17196405, 17406411, 18167404		

**Table 2:** Specifications for physical and chemical analysis.

Parameter	Method/Device	Specification
Appearance of solution	Visually by the unaided eye against a black and a white background	Clear, no color change, free from visible particles, no gas formation
pH value	pH Meter: SevenEasy, Mettler Toldeo, Columbus, OH, USA pH electrode: InLab Expert Pro, Mettler Toldeo, Columbus, OH, USA	Change in pH value < 0.4
UV-Vis absorption	UV-Vis Spectrophotometer: Evolution 201, Thermo Fisher Scientific, Waltham, USA Software: Insight 2 Software, Thermo Fisher Scientific, Waltham, MA, USA quartz cuvette Suprasil^®^ 3.5 mL, Hellma, Müllheim, Germany	Change in UV spectra < 0.04 at 420 nm Change in UV spectra < 0.01 at 550 nm
Particles > 10 µm	Particle counter, SVSS-C, PAMAS Partikelmess- und Analysesysteme GmbH, Rutesheim, Germany	≤25 particles/mL (according to European Pharmacopoeia 10.0)
Particles > 25 µm	Particle counter, SVSS-C, PAMAS Partikelmess- und Analysesysteme GmbH, Rutesheim, Germany	≤3 particles/mL (according to European Pharmacopoeia 10.0)
Drug concentration	Ultimate 3000 HPLC system, Thermo Fisher Scientific, Waltham, MA, USA: pump (LPG-3400SD) autosampler (WPS-3000TSL) column oven (TCC-3000SD) solvent rack (SR-3000) diode array detector (DAD-3000) Chromeleon 7.2 SR4 software	Drug concentration: 90–110% of the original concentration

**Table 3:** Physicochemical compatibility of clonidine.

**Binary Combinations**	
Clonidine hydrochloride, Amiodarone hydrochloride	Physically incompatible (precipitate)
Clonidine hydrochloride, Dihydralazine mesilate	Compatible (24 h)
Clonidine hydrochloride, Furosemide sodium	Physically incompatible (pH value decrease)
Clonidine hydrochloride, Levosimendan	Compatible (6 h)
Clonidine hydrochloride, Metamizole sodium	Compatible (24 h)
Clonidine hydrochloride, Milrinone	Compatible (24 h)
Clonidine hydrochloride, Urapidil	Compatible (24 h)
Clonidine hydrochloride, Verapamil hydrochloride	Compatible (24 h)
**Ternary Combinations**	
Clonidine hydrochloride, Metamizole sodium, Verapamil hydrochloride	Compatible (24 h)
Clonidine hydrochloride, Metamizole sodium, Urapidil	Compatible (6 h)
Clonidine hydrochloride, Urapidil, Verapamil hydrochloride	Compatible (24 h)

## Data Availability

The data presented in this study are available on request from the corresponding author.

## Additional Information

This work was presented in part at the ASHP Midyear Clinical Meeting and Exhibition in Anaheim, CA, 2–6 December 2018.

## Appendix A

**Table A1:** Composition of Clonidine hydrochloride 30 µg/mL produced by Pharmacy Department Erlangen University Hospital, Erlangen, Germany. The ingredients of the drug are identical to the ingredients of Paracefan^®^, Catapressan^®^ [7,46].

Ingredients	Formula
Clonidine hydrochloride, active pharmaceutical ingredient	1.5 mg
Sodium chloride, osmotic agent	0.45 mg
Hydrochloric acid 0.1 N, pH modifier	Quantum satis
Water for injection, solvent	To 50.0 mL

**Table A2:** HPLC applications: two separation methods for the quantification of clonidine hydrochloride were developed. Method I was used to separate clonidine hydrochloride from all combination partners of the study, except for metamizole. Method II was used to separate clonidine hydrochloride from a degradation product of metamizole sodium.

HPCL Method	HPLC Column	Mobile Phase	Flow Rate	Autosampler Temperature	Column Temperature	Detection	Retention Time	Dilution of Sample
Clondine I	LiChrospher^®^ 100, RP-18 endcapped (5 µm) LiChroCART^®^ 150-4.6, Lot TA5020833, Merck KGaA, Darmstadt, Germany	Acetonitrile (70 parts) NaH_2_PO_4_ buffer, pH 7.0, 1% triethylamine (30 parts)	1.0 mL/min	15 °C	40 °C	220 nm	3.1 min	Undiluted
Clonidine II	ACE Excel 3 C18-Amide^®^, 100 × 4.6 mm, Lot V13-7902, Advanced Chromatography Technologies Ltd., Aberdeen, Schottland	Acetonitrile (35 parts) NaClO_4_ buffer, pH 3.0, 2% triethylamine (65 parts)	1.0 mL/min	15° C	20 °C	220 nm	3.3 min	Undiluted
Dihydralazine	Roc Cyano 3 µm^®^, 150 × 3.0 mm, Lot 170212P, Restek, Bad Homburg, Germany	Acetonitrile (24 parts), sodiumlaurylsulfate 1%, tetrabutyl-ammonium-bromide 3% buffer, pH 3.0, (76 parts)	0.9 mL/min	4 °C	24 °C	230 nm	10.9 min	Undiluted
Levosimendan	Kinetex^®^ 2.6 µm Phenyl-Hexyl 100Å, 150 × 2.1 mm, Lot 5602-0149, Phenomenex, Torrance, CA, USA	Methanol (50 parts) K_2_HPO_4_ buffer, pH 6.8 (50 parts)	0.2 mL/min	10 °C	24 °C	374 nm	4.1 min	Undiluted
Metamizole	Kinetex^®^ 2.6 µm Phenyl-Hexyl 100Å, 150 × 2.1 mm, Lot 5602-0149, Phenomenex, Torrance, CA, USA	Methanol (50 parts) K_2_HPO_4_ buffer, pH 6.8 (50 parts)	0.2 mL/min	4 °C	24 °C	254 nm	2.4 min	1:100 (0.9% sodium-chloride)
Milrinone	Kinetex^®^ 2.6 µm Phenyl-Hexyl 100Å, 150 × 2.1 mm, Lot 5602-0149, Phenomenex, Torrance, CA, USA	Methanol (50 parts) K_2_HPO_4_ buffer, pH 6.8 (50 parts)	0.2 mL/min	4 °C	24 °C	338 nm	2.9 min	Undiluted
Urapidil	LiChrospher^®^ 100, RP-18 endcapped (5 µm) LiChroCART^®^ 150-4.6, Lot TA5020833, Merck KGaA, Darmstadt, Germany	Acetonitrile (70 parts) NaH_2_PO_4_ buffer, pH 7.0, 1% triethylamine (30 parts)	1.0 mL/min	15 °C	40 °C	230 nm	3.6 min	Undiluted
Verapamil	Kinetex^®^ 2.6 µm Phenyl-Hexyl 100Å, 150 × 2.1 mm, Lot 5602-0149, Phenomenex, Torrance, CA, USA	Methanol (70 parts) K_2_HPO_4_ buffer, pH 6.8 (30 parts)	0.22 mL/min	4 °C	24 °C	278 nm	10.9 min	Undiluted

**Table A3:** Physical compatibility of binary admixtures.

Drug Combinations, Nominal Initial Concentration (mg/mL)	Time Elapsed after Mixing (h)						
	0	0.5	1	2	4	6	24
Clonidine hydrochloride 4.5 µg/mL; Amiodarone hydrochloride 3 mg/mL	Complies with specifications	Complies with specifications	Complies with specifications	Complies with specifications	Complies with specifications	Complies with specifications	Visible, white precipitate particles ≥ 10 µm/mL: 25; 31 particles ≥ 25 µm/mL: 21; 30
Clonidine hydrochloride 15 µg/mL; Amiodarone hydrochloride 3 mg/mL	Particles ≥ 10 µm/mL: 27; 33	Particles ≥ 10 µm/mL: 28; 32	Particles ≥ 10 µm/mL: 25; 28	Particles ≥ 10 µm/mL: 26; 30	Particles ≥ 10 µm/mL: 23; 27	Particles ≥ 10 µm/mL: 25; 28	Visible, white precipitate particles ≥ 10 µm/mL: 27; 33 particles ≥ 25 µm/mL: 27; 22
Clonidine hydrochloride 4.5 µg/mL; Amiodarone hydrochloride 12 mg/mL	Particles ≥ 10 µm/mL: 33; 41	Particles ≥ 10 µm/mL: 35; 37	Particles ≥ 10 µm/mL: 33; 37	Particles ≥ 10 µm/mL: 32; 42	Particles ≥ 10 µm/mL: 32; 45	Particles ≥ 10 µm/mL: 119; 74	Visible, white precipitate particles ≥10 µm/mL: 25,313; 58,219 particles ≥ 25 µm/mL: 113; 117 420 nm: +0.269; +0.254 550 nm: +0.317; +0.280
Clonidine hydrochloride 15 µg/mL; Amiodarone hydrochloride 12 mg/mL	Particles ≥ 10 µm/mL: 25; 46	Particles ≥ 10 µm/mL: 22; 35	Particles ≥ 10 µm/mL: 20; 40	Particles ≥ 10 µm/mL: 26; 38	Particles ≥ 10 µm/mL: 24; 51	Particles ≥ 10 µm/mL: 40; 50	Visible, white precipitate particles ≥ 10 µm/mL: 10,699; 16,821 particles ≥ 25 µm/mL: 22; 13 420 nm: +0.067; +0.068 550 nm: +0.063; +0.067
Clonidine hydrochloride 4.5 µg/mL; Dihydralazine mesilate 0.25 mg/mL	Complies with specifications	Complies with specifications	Complies with specifications	Complies with specifications	Complies with specifications	Complies with specifications	Complies with specifications
Clonidine hydrochloride 15 µg/mL; Dihydralazine mesilate 0.25 mg/mL	Complies with specifications	Complies with specifications	Complies with specifications	Complies with specifications	Complies with specifications	Complies with specifications	Complies with specifications
Clonidine hydrochloride 4.5 µg/mL; Dihydralazine mesilate 0.75 mg/mL	Complies with specifications	Complies with specifications	Complies with specifications	Complies with specifications	Complies with specifications	Complies with specifications	Complies with specifications
Clonidine hydrochloride 15 µg/mL; Dihydralazine mesilate 0.75 mg/mL	Complies with specifications	Complies with specifications	Complies with specifications	Complies with specifications	Complies with specifications	Complies with specifications	Complies with specifications
Clonidine hydrochloride 4.5 µg/mL; Furosemide sodium 0.64 mg/mL	Complies with specifications	Complies with specifications	Complies with specifications	Complies with specifications	Complies with specifications	Complies with specifications	Complies with specifications
Clonidine hydrochloride 15 µg/mL; Furosemide sodium 0.64 mg/mL	Complies with specifications	Complies with specifications	Complies with specifications	pH: −0.5; −0.4	pH: −0.6; −0.5	pH: −0.7; −0.5	pH: −0.7; −0.5
Clonidine hydrochloride 4.5 µg/mL; Furosemide sodium 5.34 mg/mL	Complies with specifications	Complies with specifications	Complies with specifications	Complies with specifications	Complies with specifications	pH: −0.4; −0.4	pH: −0.9; −0.7
Clonidine hydrochloride 15 µg/mL; Furosemide sodium 5.34 mg/mL	Complies with specifications	Complies with specifications	Complies with specifications	Complies with specifications	pH: −0.4; −0.5	pH: −0.5; −0.4	pH: −1.1; −0.9
Clonidine hydrochloride 4.5 µg/mL; Levosimendan 12.5 µg/mL	Complies with specifications	420 nm: +0.174; +0.146	420 nm: +0.183; +0.152	420 nm: +0.197; +0.157	420 nm: +0.202; +0.167	420 nm: +0.195; +0.178	420 nm: +0.250; +0.217
Clonidine hydrochloride 15 µg/mL; Levosimendan 12.5 µg/mL	Complies with specifications	420 nm: +0.043; +0.045	420 nm: +0.054; +0.069	420 nm: +0.093; +0.100	420 nm: +0.107; +0.094	420 nm: +0.107; +0.100	420 nm: +0.132; +0.157
Clonidine hydrochloride 4.5 µg/mL; Metamizole sodium 25 mg/mL	Complies with specifications	Complies with specifications	pH: +0.4; +0.4	pH: +0.4; +0.5	pH: +0.4; +0.4	pH: +0.4; +0.4	pH: +0.4; +0.3 420 nm: +0.099; +0.110
Clonidine hydrochloride 15 µg/mL; Metamizole sodium 25 mg/mL	Complies with specifications	Complies with specifications	pH: +0.5; +0.3	pH: +0.5; +0.4	pH: +0.3; +0.4	pH: +0.3; +0.3 420 nm: +0.035; +0.053	pH: +0.3; +0.4 420 nm: +0.105; +0.139
Clonidine hydrochloride 4.5 µg/mL; Metamizole sodium 50 mg/mL	Complies with specifications	Complies with specifications	Complies with specifications	Complies with specifications	Complies with specifications	pH: +0.3; +0.4 420 nm: +0.050; +0.042	pH: +0.4; +0.5 420 nm: +0.131; +0.133
Clonidine hydrochloride 15 µg/mL; Metamizole sodium 50 mg/mL	Complies with specifications	Complies with specifications	Complies with specifications	Complies with specifications	Complies with specifications	pH: +0.4; +0.4 420 nm: +0.040; +0.076	pH: +0.4; +0.3 420 nm: +0.126; +0.175
Clonidine hydrochloride 4.5 µg/mL; Milrinone 0.1 mg/mL	Complies with specifications	Complies with specifications	Complies with specifications	Complies with specifications	Complies with specifications	Complies with specifications	Complies with specifications
Clonidine hydrochloride 15 µg/mL; Milrinone 0.1 mg/mL	Complies with specifications	Complies with specifications	Complies with specifications	Complies with specifications	Complies with specifications	Complies with specifications	Complies with specifications
Clonidine hydrochloride 4.5 µg/mL; Urapidil 1 mg/mL	Complies with specifications	Complies with specifications	Complies with specifications	Complies with specifications	Complies with specifications	Complies with specifications	Complies with specifications
Clonidine hydrochloride 15 µg/mL; Urapidil 1 mg/mL	Complies with specifications	Complies with specifications	Complies with specifications	Complies with specifications	Complies with specifications	Complies with specifications	Complies with specifications
Clonidine hydrochloride 4.5 µg/mL; Urapidil 2.5 mg/mL	Complies with specifications	Complies with specifications	Complies with specifications	Complies with specifications	Complies with specifications	Complies with specifications	Complies with specifications
Clonidine hydrochloride 15 µg/mL; Urapidil 2.5 mg/mL	Complies with specifications	Complies with specifications	Complies with specifications	Complies with specifications	Complies with specifications	Complies with specifications	Complies with specifications
Clonidine hydrochloride 4.5 µg/mL; Verapamil hydrochloride 0.5 mg/mL	Complies with specifications	Complies with specifications	Complies with specifications	Complies with specifications	Complies with specifications	Complies with specifications	Complies with specifications
Clonidine hydrochloride 15 µg/mL; Verapamil hydrochloride 0.5 mg/mL	Complies with specifications	Complies with specifications	Complies with specifications	Complies with specifications	Complies with specifications	Complies with specifications	Complies with specifications

**Table A4:** Physical compatibility of ternary admixtures.

Drug Combinations, Nominal Initial Concentration (mg/mL)	Time Elapsed after Mixing (h)						
	0	0.5	1	2	4	6	24
Clonidine hydrochloride 10 µg/mL; Metamizole sodium 33 mg/mL; Urapidil 1.67 mg/mL	Complies with specifications	Complies with specifications	Complies with specifications	Complies with specifications	Complies with specifications	420 nm: +0.040; +0.043	420 nm: +0.123; +0.140
Clonidine hydrochloride 10 µg/mL; Metamizole sodium 33 mg/mL; Verapamil hydrochloride 0.33 mg/mL	Complies with specifications	Complies with specifications	Complies with specifications	Complies with specifications	Complies with specifications	420 nm: +0.042; +0.043	420 nm: +0.109; +0.122
Clonidine hydrochloride 10 µg/mL; Urapidil 1.67 mg/mL; Verapamil hydrochloride 0.33 mg/mL	Complies with specifications	Complies with specifications	Complies with specifications	Complies with specifications	Complies with specifications	Complies with specifications	Complies with specifications

**Table A5:** Chemical compatibility of binary admixtures.

Drug Combinations, Nominal Initial Concentration (mg/mL)	Time Elapsed after Mixing (h)						
	0	0.5	1	2	4	6	24
Clonidine hydrochloride 4.5 µg/mL; Dihydralazine mesilate 0.25 mg/mL	100.00 100.00	99.64; 99.84 98.32; 99.57	99.59; 98.68 98.32; 100.43	99.05; 99.49 98.74; 100.43	99.20; 99.44 99.16; 100.00	98.89; 99.60 98.32; 100.85	99.52; 99.42 98.73; 100.85
Clonidine hydrochloride 5 µg/mL; Dihydralazine mesilate 0.25 mg/mL	100.00 100.00	99.80; 98.69 102.08; 101.67	100.41; 99.73 102.08; 101.67	99.79; 99.17 101.67; 101.67	100.28; 99.66 102.08; 101,67	100.16; 99.00 101.67; 101.67	100.02; 98.92 101.67; 101.67
Clonidine hydrochloride 4.5 µg/mL; Dihydralazine mesilate 0.75 mg/mL	100.00 100.00	100.05; 99.89 100.26; 99.74	99.75; 99.17 100.00; 100.00	100.46; 99.63 100.00; 100.13	99.59; 99.74 100.26; 100.26	99.77; 99.96 100.51; 100.51	99.27; 99.93 98.85; 98.85
Clonidine hydrochloride 15 µg/mL; Dihydralazine mesilate 0.75 mg/mL	100.00 100.00	100.89; 99.28 99.74; 99.49	100.06; 99.62 99.61; 99.11	99.24; 99.22 99.87; 98.73	99.12; 99.06 100.77; 98.60	99.27; 99.35 93.62; 93.76	99.61; 99.58 92.35; 92.23
Clonidine hydrochloride 4.5 µg/mL; Levosimendan 12.5 µg/mL	100.00 100.00	100.11; 99.72 100.00; 100.00	100.37; 99.98 100.00; 100.00	100.19; 100.02 100.00; 100.00	100.41; 100.02 100.00; 100.00	100.13; 99.74 92.86; 91.67	99.89; 99.51 85.71; 83.33
Clonidine hydrochloride 15 µg/mL; Levosimendan 12.5 µg/mL	100.00 100.00	99.99; 100.30 100.00; 100.00	100.12; 100.04 100.00; 100.00	99.66; 100.05 100.00; 100.00	100.12; 99.93 100.00; 100.00	100.12; 99.93 92.86; 92.31	100.12; 100.15 85.71; 84.62
Clonidine hydrochloride 4.5 µg/mL; Metamizole sodium 25 mg/mL	100.00 100.00	100.09; 99.86 99.19; 100.00	99.75; 99.80 95.55; 94.94	99.95; 99.77 95.14; 94.09	98.93; 98.68 95.55; 94.09	98.88; 98.02 94.33; 93.67	99.09; 98.84 93.93; 94.94
Clonidine hydrochloride 15 µg/mL; Metamizole sodium 25 mg/mL	100.00 100.00	100.12; 100.10 94.40; 93.03	99.98; 99.93 92.67; 93.44	99.49; 99.61 93.97; 93.44	99.32; 99.33 92.24; 93.03	98.50; 98.50 92.67; 91.80	98.39; 98.39 92.24; 90.98
Clonidine hydrochloride 4.5 µg/mL; Metamizole sodium 50 mg/mL	100.00 100.00	99.26; 99.86 99.20; 100.00	99.72; 100.07 97.38; 98.02	99.63; 99.88 97.59; 96.44	99.77; 99.93 96.98; 96.44	99.58; 100.07 96.58; 96.25	99.63; 100.02 95.37; 93.87
Clonidine hydrochloride 15 µg/mL; Metamizole sodium 50 mg/mL	100.00 100.00	99.92; 99.92 98.57; 98.59	99.75; 99.78 96.33; 96.37	99.64; 99.60 96.94; 96.37	98.09; 98.17 95.72; 95.77	98.07; 98.15 96.33; 95.16	95.53; 95.71 93.08; 94.35
Clonidine hydrochloride 4.5 µg/mL; Milrinone 0.1 mg/mL	100.00 100.00	99.98; 99.78 100.00; 99.00	99.91; 99.76 100.00; 101.00	99.87; 99.78 99.01; 99.00	99.89; 99.74 99.01; 100.00	99.85; 99.76 99.01; 100.00	99.80; 99.52 100.00; 101.00
Clonidine hydrochloride 15 µg/mL; Milrinone 0.1 mg/mL	100.00 100.00	99.99; 99.13 101.02; 101.02	100.07; 99.15 101.02; 101.02	99.84; 99.21 101.02; 100.00	99.93; 99.57 101.02; 100.00	99.92; 99.63 101.02; 101.02	99.77; 99.62 102.04; 101.02
Clonidine hydrochloride 4.5 µg/mL; Urapidil 1 mg/mL	100.00 100.00	99.73; 99.61 99.90; 99.50	99.82; 99.96 100.00; 99.70	99.93; 99.85 99.49; 98.69	99.91; 99.91 99.80; 99.40	99.89; 100.04 99.19; 98.59	100.09; 99.85 98.99; 99.90
Clonidine hydrochloride 15 µg/mL; Urapidil 1 mg/mL	100.00 100.00	99.97; 99.36 100.89; 99.80	100.29; 99.36 100.89; 100.30	100.11; 99.69 100.89; 100.40	100.12; 98.55 100.89; 100.00	100.06; 98.41 100.50; 100.00	99.83; 98.66 100.79; 100.70
Clonidine hydrochloride 4.5 µg/mL; Urapidil 2.5 mg/mL	100.00 100.00	100.40; 99.91 99.76; 100.45	100.36; 99.46 98.82; 99.92	100.07; 99.76 100.04, 99.59	100.31; 100.39 100.08; 101.03	100.25; 99.76 99.92; 100.25	100.31; 99.70 100.24; 100.33
Clonidine hydrochloride 15 µg/mL; Urapidil 2.5 mg/mL	100.00 100.00	99.00; 100.27 100.00; 100.00	98.79; 100.30 99.31; 99.06	99.67; 100.51 99.88; 100.00	99.28; 100.51 99.84; 99.14	99.41; 100.29 98.90; 99.55	100.14; 100.43 99.88; 99.84
Clonidine hydrochloride 4.5 µg/mL; Verapamil hydrochloride 0.5 mg/mL	100.00 100.00	100.18; 100.00 99.82; 100.72	100.11; 99.89 100.36; 99.28	100.09; 99.91 99.82; 100.18	100.07; 99.96 100.09; 100.72	100.02; 99.89 100.90; 99.82	100.09; 99.84 99.82; 100.18
Clonidine hydrochloride 15 µg/mL; Verapamil hydrochloride 0.5 mg/mL	100.00 100.00	100.01; 100.01 99.64; 101.26	100.02; 99.96 99.82; 99.64	100.03; 99.98 99.11; 98.56	100.02; 99.99 99.46, 99.82	100.01; 99.99 99.82; 100.54	99.40; 99.89 99.46; 100.36

**Table A6:** Chemical compatibility of ternary admixtures.

Drug Combinations, Nominal Initial Concentration (mg/mL)	Time Elapsed after Mixing (h)						
	0	0.5	1	2	4	6	24
Clonidine hydrochloride 10 µg/mL; Metamizole sodium 33 mg/mL; Urapidil 1.67 mg/mL	100.00 100.00 100.00	99.89; 100.06 99.68; 99.64 99.82; 99.35	99.89; 99.92 96.85; 97.83 99.35; 98.82	100.00; 100.14 95.90; 93.48 97.22; 97.83	100.15; 100.31 93.69; 93.84 97.40; 96.77	100.21; 100.30 93.06; 92.75 96.28; 95.65	100.31; 100.53 92.11; 90.22 89.25; 87.66
Clonidine hydrochloride 10 µg/mL; Metamizole sodium 33 mg/mL; Verapamil hydrochloride 0.33 mg/mL	100.00 100.00 100.00	100.01; 99.99 96.98; 94.69 99.12; 99.42	99.96; 99.98 95.77; 93.44 97.65; 98.26	99.94; 99.86 95.47; 92.81 98.24; 97.38	99.97; 99.64 90.94; 92.50 98.24; 96.22	99.98; 99.80 91.54; 90.94 97.35; 96.80	99.95; 97.64 90.33; 90.94 97.06; 95.93
Clonidine hydrochloride 10 µg/mL; Urapidil 1.67 mg/mL; Verapamil hydrochloride 0.33 mg/mL	100.00 100.00 100.00	99.54; 99.78 99.71; 100.06 98.53; 100.00	99.76; 99.98 100.58; 100.35 100.00; 99.71	99.70; 99.82 100.06; 100.41 99.41; 100.00	99.48; 99.85 99.94; 99.47 100.29; 99.42	99.50; 99.84 99.19; 100.12 99.71; 99.14	99.25; 99.75 99.88; 99.59 99.41; 99.42
